# A study on the relationship between compensation gap within the top management team and corporate performance: An empirical research based on the moderation effect of fairness preference

**DOI:** 10.3389/fpsyg.2022.916832

**Published:** 2022-11-24

**Authors:** Xi Wang, Xue Chen, Haoqian Zhou, Xiangbo You

**Affiliations:** Business School, Tianjin University of Finance and Economics, Tianjin, China

**Keywords:** tournament theory, fairness preference, the compensation gap within the top management team (TMT), FS model, social preference

## Abstract

This study explores the relationship between the compensation gap within the top management team (TMT) and corporate performance. We focus on how the fairness preference of the TMT moderates this relationship. The existing researches on the relationship between the compensation gap within the TMT and corporate performance are inconclusive. The reason may be that the traditional tournament theory is based on the hypothesis of self-interest preference of homo economicus. In the research, the fairness preference theory is added to the traditional tournament model, and a more realistic tournament model considering fairness preference is constructed. Based on the analysis of the theoretical model and the empirical regression analysis of the panel data of 733 non-financial A-share listed companies in Shanghai and Shenzhen stock markets from 2014 to 2020, we draw the following main conclusions: (1) There is an inverted U-shaped relationship between the TMT compensation gap and the corporate performance. Within the optimal compensation gap, there is a significant positive correlation. The larger the compensation gap, the better the corporate performance will be. When the optimal compensation gap is exceeded, there is a significant negative correlation. The larger the compensation gap, the worse the corporate performance will be. (2) The fairness preference of the TMT will weaken the correlation between the TMT compensation gap and corporate performance. Within the optimal compensation gap, the fairness preference will weaken the positive relationship between them, and when it exceeds the optimal compensation gap, the fairness preference will also weaken the negative relationship between them.

## Introduction

In the context of asymmetric information, how to design an effective incentive mechanism to motivate managers to take action and maximize the principal’s utility has become one of the focus issues in theory and practice. Under symmetric information, neoclassical economic theory advocates that marginal output determines the level of compensation. However, in the case of asymmetric information, managers can attribute low profits to unfavorable exogenous influences, thus evading the accusation of the principal and causing the “moral hazard” problem. Lazear and Rosen (1981) proved that if the agent’s performance is relevant, the rank-order tournaments can eliminate more uncertain factors, to make the principal’s judgment on the manager’s effort level more accurate. When greater rewards are provided for high performers, tournament theory suggests that improved effort and performance can be attained (Lambert et al., 1993). The introduction of performance-related pay systems typically leads to an increase in the dispersion of wages. Several empirical studies on the relationship between the compensation gap of the top management team (TMT) and corporate performance have not reached uniform conclusions. Some studies show that there is a linear positive relationship between the compensation gap and corporate performance, which supports the tournament theory (Main et al., 1993; Lin et al., 2003; Lallemand et al., 2004; Lu R., 2007; Mahy et al., 2011; Li et al., 2012; Huo et al., 2019; Niu et al., 2019; Huang et al., 2022). Other empirical studies have come to the opposite conclusion, arguing that the expansion of the compensation gap will damage corporate performance, and there is a linear negative relationship between them (Cowherd and Levine, 1992; Siegel and Hambrick, 2005; Zhang, 2007, 2008; Fredrickson et al., 2010; Liu et al., 2017; Eidd and Abou-Moghlie, 2021; Li and Jiao, 2021). In addition, others provide evidence that there is a nonlinear relationship between the compensation gap and corporate performance. For example, Bingley and Eriksson’s (2001) research on Danish enterprises and Chen and Zhang’s (2010) theoretical and empirical study found an inverted U-shaped relationship between them. In this nonlinear relationship, the positive relationship, in reality, indicates that it is in the nonoptimal rising stage, and the negative relationship indicates that it is in the nonoptimal falling stage. Some scholars have also demonstrated the opposite positive U-shaped relationship (Grund and Westergaard-Nielsen, 2008; Hu and Fu, 2018).

Despite a growing body of research, our knowledge of the issue remains woefully limited. The inconclusive conclusions make us confused. Should we increase or reduce the compensation gap of the TMT in the pay structure design? It is essential to explore the relationship between the compensation gap within the TMT and corporate performance. We focus on the internal influence mechanism of the TMT compensation gap on corporate performance. The reason for the inconclusive conclusion of empirical studies may be that the traditional tournament theory is only limited to the hypothesis of homo economicus without considering the fairness preference of agents. Behavioral experiments such as ultimatum game, gift exchange game, trust game, and public good game show that the pursuit motivation of individual economic interests alone can not fully explain the behavior of participants. The pursuit of “fairness” is also an important explanatory factor of their behavior, that is, individuals have fairness preferences. Scholars have gradually begun to pay attention to the incentive effect of agents’ non-pure self-interest preference, but there are few studies on the application of fairness preference to tournament theory, most of which are just model construction and theoretical analysis. There are few studies using the data of listed companies to empirically test the moderating effect of fairness preference in real economic operations. Based on the traditional tournament model, we take fairness preference into account discussing a theoretical tournament model on the fairness preference of agents. We also conduct an empirical test with 733 nonfinancial A-share listed companies in Shenzhen and Shanghai stock markets from 2014 to 2020 as research samples to investigate the relationship between the compensation gap within the TMT and the corporate performance under fairness preference.

The study is structured as follows: following the study pattern, the section “Introduction” presents the research background and the purpose of the study. Section “Literature review” presents the theoretical basis and a literature review. Section “Theoretical analysis and hypotheses” introduces the theoretical model deduction and develops the study hypotheses. Section “Methodology and results” presents the study sample and research methodology. The findings of the study are also presented in the section. Section “Discussion” compare the results with other studies. The “Conclusion” section summarizes the study conclusions. Section “Implications” concludes the study with its limitations, future directions, and management implications.

## Literature review

### Tournament theory

Rank-order tournaments or tournament theory is a compensation system based on relative performance evaluation, which was first proposed by Lazear and Rosen (1981). The incentive mechanism proposes to rank the outputs of all participants in order and gives a promotion bonus to the participants with relatively more output, to achieve the purpose of motivating the participants to win the competition by making efforts, thereby improving the corporate performance. The basic hypotheses of the theory are: first, the success or failure of the competition depends on the comparison of the relative performance of the participants; second, the higher the overall compensation level of the management and the larger the internal compensation gap, the better the incentive effect of the mechanism; third, the compensation gap within management team should increase with the increase in the number of people participating in the competition and position levels. These hypotheses are supported by relevant studies (Bull et al., 1987; Eriksson, 1999; Conyon et al., 2001). At the same time, a potential hypothesis of tournament theory is that the agents are purely self-interested, and their utilities depend on the individual’s compensation and the corresponding cost, rather than the comparison with other participants.

Tournament theory explains the phenomenon that the compensation of senior executives increases significantly after promotion. Since it is difficult to measure the performance of senior executives and monitor their efforts, the gap between compensation levels can motivate the effective efforts of senior executives, thus promoting the consistency of interests between principals and agents and reducing agency costs. Since then, several scholars have applied this theory to the research on the salary gap of other positions within enterprises and achieved a lot of results. The main contributions of the theory are: first, when the risk preference of participants is risk neutral, the system can achieve the same resource allocation efficiency as the marginal output system; second, it is easier to observe relative marginal outputs sequentially than to directly measure the marginal outputs of each player, especially if monitoring costs are high. It can not only greatly reduce the monitoring cost of agents but also achieve the ideal result of motivating the efforts of participants (Lazear and Rosen, 1981; Rosen, 1986). In addition, the promotion bonus, that is, the compensation gap is the attraction and encouragement for managers to participate in the ranking competition, which can motivate competitors consciously make greater efforts and reduce the necessity of enterprise monitoring.

According to the tournament theory, enterprises should increase the compensation gap between position levels in order to reduce the principal-agent cost and improve corporate performance. Leonard (1990); Lambert et al. (1993), and Eriksson (1999) found that when the internal compensation gap of senior management remains unchanged, simply increasing the compensation level of senior executives could not improve their efforts, which supports the theoretical proposition that the key to encouraging managers to improve performance is the internal compensation gap. The research of Tsou and Liu (2005) believes that when the compensation gap in the enterprise is small, the turnover rate of employees is high, which also supports the design of increasing the compensation gap.

### Fairness preference theory

In the 1980s, many classical game experiments, such as the ultimatum game experiment (Güth et al., 1982), dictator game experiment (Andreoni and Miller, 1993; Forsythe et al., 1994), trust game experiment (Berg et al., 1995), gift exchange game experiment (Akerlof, 1982), and public good game experiment (Marwell and Ames, 1979; Fehr and Gächter, 2000) strongly demonstrated the existence of social preferences including fairness preference, reciprocity preference, and altruism preference. Social preference theory has relatively complete and mature economic models, such as the fairness preference model (result oriented), reciprocity preference model (motivation oriented), and social welfare preference model (altruism oriented). It has extensive influence and strong academic vitality, among which fairness preference theory is particularly attractive (Chen et al., 2012).

Fairness preference, also known as inequality aversion preference, is a social preference oriented by the result of income distribution. It assumes that participants only pay attention to the fairness of the result, regardless of the goodwill of the opponent. Under this preference, participants have the motivation to narrow the income gap with others. The proposals of the classical FS model and BO model indicate the maturity of fairness preference theory (Loewenstein et al., 1989; Bolton, 1991).

The FS model was proposed by Fehr and Schmidt (1999). According to the model, income inequality will hinder the individual’s utility level. When people find that their income is lower than others through comparison, there is a loss of utility due to disadvantage inequality or jealousy. When they find that their income is higher than others, they will feel the utility loss due to advantage inequality or sympathy. The results of the model show that when the income gap between the participant and others is zero, their utility is maximized, that is, individuals will strive to pursue the indifference of income.

The BO model was proposed by Bolton and Ockenfels (2000), also known as the ERC (equity, reciprocity, and competition) model. The model is similar to the FS model but depicts the environmental background of incomplete information and uses a nonlinear form. It holds that individual utility is not only influenced by absolute income but also a function of relative income. The results of the model show that participants will strictly prefer the average income value of the reference group, that is, they will make their income share tend to the average level through practical actions.

Both the FS model and BO model believe that in the case of fairness preference, the equal income of participants is the optimal solution. The difference between the two is that the FS model measures the absolute income gap between individuals, while the BO model explores the relative share of individual income in the overall income. Among them, the FS model has been recognized and widely used by many scholars because it can more reasonably explain the behavior results in various game experiments, and the model structure is simple and easy to apply.

### The compensation gap within the top management team and the corporate performance

Scholars in China and abroad have carried out a lot of research on whether the compensation gap within the TMT can have a positive effect on corporate performance, but the conclusions are not consistent. A considerable number of studies have found that the compensation gap within the TMT positively affects corporate performance, which is consistent with the opinions of tournament theory. Eriksson (1999) conducted empirical research on 210 enterprises in Denmark and pointed out that the widening of the compensation gap between CEOs and submanagers contributed to the improvement of sales profit margin, and its contribution was about 4–5%. Lee et al. (2008) used 10 years’ data of American listed companies and found that the compensation difference within the TMT could positively predict corporate performance, and this relationship was more significant in an effective governance structure. Xu et al. (2016) pointed out in the research based in China that the positive correlation between the two existed only in non-state-owned enterprises. Heyman’s (2005) research on the data of 10,000 managers showed that executive compensation dispersion positively affected profits, and the results of Sanchez-Marin and Baixauli-Soler (2015) using Spanish data also supported this opinion. Burns et al. (2017) used multinational samples to show that the trophy structure, that is, the compensation gap between CEOs and other senior executives, varied with national cultural characteristics and was positively related to corporate performance. Lin et al. (2003) empirically found that the larger the compensation gap between the CEO and other senior executives was, the higher the corporate future performance. Lu H. (2007, 2009) and Liu et al. (2011) reached the same conclusion. The research of Zhang and Li (2018) showed that the compensation gap of the senior executive team could send a positive signal of the corporate performance to market investors and reduce the bond issuance spreads. Ma et al. (2020) proposed that local tournaments could promote corporate performance, and this result was equally applicable to CEOs and CFOs. Zhong et al. (2021) also proved internal vertical compensation gap promotes firm innovation performance, but CEO’s power weakens the positive effect between them.

Second, some theoretical and empirical studies believe that the expansion of the compensation gap will be harmful to organizational performance. Lazear (1989) further proposed based on the tournament model that sabotage was a basic feature of the tournament system. When the employees’ behaviors can influence each other, they may sabotage in order to win. This behavior has a double negative impact on corporate performance because it damages others’ output and their own output at the same time and becomes more intense with the increase in promotion bonuses (Harbring and Irlenbusch, 2008). A study of executive compensation levels by O’Reilly et al. (1988) showed that the results did not conform to the tournament theory but strongly supported the social comparison theory. Fredrickson et al. (2010) also supported the inverse relationship between executive compensation dispersion and corporate performance from the perspective of social comparison theory. Carpenter and Sanders (2004) found that the compensation gap between CEOs and the senior executive team had a negative effect on performance in the coming years. Siegel and Hambrick (2005) further believed that this situation would be more serious in high-tech enterprises due to the requirements for the interdependence of the TMT members. Zhang (2007, 2008) and Zhang and Li (2007) focused on the compensation gap of core members of the senior executive team of listed companies, and the results showed that it had a limited impact on corporate performance. Mei and Zhao (2016) pointed out that both vertical and parallel compensation gaps of senior executives would increase the turnover rate of vice presidents, further reducing corporate performance.

With the further development of research, other scholars believe that there is a nonlinear inverted U-shaped relationship between the compensation gap and corporate performance. In fact, Lazear and Rosen (1981) and Grund and Sliwka (2005) have proposed the theoretical value of the optimal compensation gap in the analysis of the tournament model. Lin et al. (2003) proved that there was a linear relationship between the CEO compensation gap and corporate performance, but they believed that this was only because the compensation gap during the investigation period was far from the optimal value, and its negative effects had not yet appeared. The relationship between the compensation gap and future performance was likely to be a quadratic curve. Qin (2009) deduced that the relationship between the compensation gap within the TMT and the expected return of the enterprise was positive at first and then negative through the reestablishment of the multiagent compensation contract model. The empirical research found that the degree of compensation inequity had not yet approached the inverted U-shaped inflection point. Chen and Zhang (2010) took destructive behavior into account in the tournament model, and Huang (2012) further deduced the tournament model. They both concluded that the theoretical relationship between the compensation gap and corporate performance is inverted U-shaped. The former also investigated the interval effect between them through empirical methods, which is in line with the results of Bingley and Eriksson (2001) based in Denmark. Chen et al. (2019) proposed that there is a significant inverted U-shaped relationship between the compensation gap and corporate performance, and the correlation between the two is more significant in enterprises with higher performance.

In addition, a few scholars have reached other different conclusions. Empirical studies by Grund and Westergaard-Nielsen (2008) and Ridge et al. (2015) supported that compensation dispersion played a positive U-shaped role in corporate performance. Similarly, Hu and Fu (2018) used the OLS method and 2SLS method to conclude that the compensation gap within the enterprise (including the senior executive team and senior executive-employee) had a U-shaped relationship with corporate accounting performance and market performance and was moderated by factors such as operational risk. The long-term study by Connelly et al. (2016) showed that the effects of compensation dispersion on the short-term performance and long-term performance of the company are completely opposite. Lu (2011) and He and Zhang (2017) believed that the positive and negative relationships between the compensation gap of the senior executive team and corporate performance were determined by the degree of risk and debt.

### Fairness preference, the compensation gap within the top management team, and the corporate performance

In recent years, some scholars have begun to introduce fairness preference into the tournament theory and have made pioneering research on incentive theory. Kräkel (2000) analyzed the effort level of agents in the tournament model based on the theory of relative exploitation and pointed out that the income comparison between agents and their colleagues was a greater motivation for agents to make efforts. Demougin and Fluet (2003) believed that the agent’s jealousy might be beneficial to the principal, and this possibility depended on the cost of performance evaluation. Grund and Sliwka (2005) integrated the FS model into the tournament model and discussed the impact of fairness preference on employees’ effort provision and corporate profits. They argued that when the bonus was given, the corporate profits in the condition of competition of fairness preference agents are higher than that in the condition of self-interest preference agents. If the bonus structure could be adjusted, the incentive effect would disappear completely and the participation effect would be dominant. Gill and Stone (2010) integrated tournament theory, fairness theory, and loss aversion theory to model the agent’s competitive behavior based on the self-value evaluation. Through the discussion of the relationship between fairness preference and self-value, it was found that one reason why tournaments were widely accepted was the formation of internal reference points of self-value evaluation. Eisenkopf and Teyssier (2013) used the game experimental method and confirmed that jealousy and loss aversion would lead agents to pay extra effort to avoid disappointment and lower returns, and some agents with loss aversion preference would greatly reduce efforts. In general, fairness preference would lead to a reduction in total utility and tournament incentive efficiency.

Chinese scholars Wei and Pu (2006) introduced the FS model into the tournament model with the behavior of sabotage. The conclusion was that fairness preference would reduce the agent’s effort provision and the behavior of sabotage. Compared with pure self-interest, the expected income of the principal with fairness preference was lower, so it was best for enterprises to implement the tournament system among agents with pure self-interest or weak fairness preference. Similar to the conclusion, Liu et al. (2014) constructed a more complex three-stage tournament model with the introduction of fairness preference and found that the change direction of effort and sabotage was the same, while the impact of fairness preference and compensation gap between them was quite opposite. The former reduced them and the latter improved them. Wei and Tang (2017) studied the effect of tournaments in the condition of heterogeneous preferences and suggested that principals carefully identified the preference types of participants because the expected profit of implementing group competitions based on heterogeneous preferences was greater than that of pooled tournaments.

## Theoretical analysis and hypotheses

### The relationship between the compensation gap and the corporate performance based on traditional tournament model

In our research, the tournament model without fairness preference (Lazear and Rosen, 1981; Grund and Sliwka, 2005; Wei and Pu, 2006) is called the traditional tournament model, which is used as the basic model and comparison of derivation, and its derivation process and conclusion are listed. Based on the potential reason for the behavior of sabotage is likely to be fairness preference, so the traditional tournament model discussed in this article does not include sabotage.

Considering the simple traditional tournament model of two-person competition, there are two homogeneous agents A and B in this model. Their output function *Q*(*e*) and cost function *C*(*e*) are exactly the same, both of which are functions of effort level *e*. The output function is *Q*(*e*_*i*_) = *F*(*e*_*i*_) + ε_*i*_, *F*(*e*_*i*_) is a concave function, *F*′ > 0, *F*′′ < 0, ε_*i*_ is independent and identically distributed; the cost function *C*(*e*_*i*_) is convex, *C*′ > 0, *C*′′ > 0, and *F*(0) = *C*(0) = 0. In the tournament between agents A and B, the amount of promotion bonus is *WD*. The winner in the tournament will receive monetary compensation *W*_*H*_, and the loser will receive monetary compensation *W*_*L*_, that is, *WD = W_*H*_−W_*L*_*. Furthermore, the probability of victory for agent *i* is $P_{i}^{H}$.

Under the pure self-interest hypothesis, the utility of the agent is only related to the reward. The agent’s utility in winning the competition is: *U^H^* = W_*H*_−C(e), while the agent’s utility in losing the competition is: *U^L^* = W_*L*_−C(e). Therefore, the expected utility of agent *i* can be expressed as:


$$E\,U_{i}=P_{i}^{H}\,U^{H}+\left(1-P_{i}^{H}\right)\,U^{L}=P_{i}^{H}\,W\,D+W_{L}-C\,\left(e_{i}\right)$$

where the probability of victory $P_{i}^{H}=p\left(Q_{i}>Q_{j}\right)$


$$=p\left(F\left(e_{i}\right)+\varepsilon_{i}>F\left(e_{j}\right)+\varepsilon_{j}\right)$$

$$=p\left(\varepsilon_{j}-\varepsilon_{i}<F\left(e_{i}\right)-F\left(e_{j}\right)\right)$$

The random variable ξ = ε_*j*_−ε_*i*_ obeys the probability distribution function G(⋅) with the probability density g(⋅), *E*ξ = 0, g(−x) = g(x), so $P_{i}^{H}=G\,\left(F\,\left(e_{i}\right)-F\,\left(e_{j}\right)\right)$, *EU*_*i*_ = *G*(*F*(*e*_*i*_)−*F*(*e*_*j*_))*WD* + *W*_*L*_−*C*(*e*_*i*_).

Under a given compensation structure, agents maximize their expected utility by choosing the degree of effort they make, that is, let $\frac{\partial E\,U_{i}}{\partial e_{i}}=0$, we get


$$\text{g}\,\left(\text{F}\,\left({\text{e}}_{\text{i}}\right)-\text{F}\,\left({\text{e}}_{\text{j}}\right)\right)\,F^{\prime }\,\left(e_{i}\right)\,W\,D-C^{\prime }\,\left(e_{i}\right)=0$$

$$\text{g}\,\left(F\,\left(e_{j}\right)-F\,\left(e_{i}\right)\right)\,F^{\prime }\,\left(e_{j}\right)\,W\,D-C^{\prime }\,\left(e_{j}\right)=0$$

From the symmetry of pure strategy Nash equilibrium, we get *e_i_ = e_j_*, then the maximization condition of the agent’s expected utility can be expressed as:


(1) $$\frac{C^{\prime }}{F^{\prime }}=\text{g}\,\left(0\right)\,W\,D$$

At this time, the probability of victory $P_{i}^{H}=G\,\left(0\right)=\frac{1}{2}$, $E\,U_{i}=\frac{1}{2}\,W\,D+W_{L}-C\,\left(e_{i}\right)$.

Equation 1 is called incentive compatibility constraint (Wei and Pu, 2006), and further derivation of e to the left of the equal sign can be obtained (C′F′)′=C″⁢F′-C′⁢F″F2′>0. When the compensation gap *WD* to the right of the equal sign expands, $\frac{C^{\prime }}{F^{\prime }}$ increases and the effort degree e also increases. This shows that the agent’s effort provision depends on the compensation gap WD. The larger the compensation gap, the more effort provision will be.

At the same time, the agent will withdraw from the competition if the expected utility is lower than the minimum reservation utility; that is, *EU*_*i*_≥*U*_0_, and in equilibrium,


(2) $$W_{L}+\frac{1}{2}\,W\,D-C\,\left(e\right)\geq U_{0}$$

Equation 2 is called participation constraint (Wei and Pu, 2006), and it can be seen that when the loser’s compensation *W*_*L*_ remains unchanged and the compensation gap *WD* increases, the effort provision will also promote.

When the agent chooses to make efforts independently, the principal’s income is *Per* = *Q*_*i*_ + *Q*_*j*_−(*W*_*H*_ + *W*_*L*_), and the expected income is


(3) $$E\,P\,e\,r=2\,F\,\left(e\right)-2\,W_{L}-W\,D$$

The principal should try to set an optimal compensation gap to maximize the expected income. According to Grund and Sliwka (2005), the participation constraint in equilibrium, that is, Equation 2 should be equal. Otherwise, the principal will reduce the loser’s compensation *W*_*L*_ and finally make the Equation 2 equal. Therefore, we can get:


(4) $$W_{L}+\frac{1}{2}\,W\,D-C\,\left(e\right)=U_{0}$$

The derivation of *WD* on both sides of Equation 4 can be obtained:


(5) $$\frac{1}{2}-C^{\prime }\,\frac{\partial e}{\partial W\,D}=0$$

From Equation 1, *C*′ = g(0)*WDF*′, and we substitute it in Equation 5 to get:


(6) $$\frac{\partial e}{\partial W\,D}=\frac{1}{2\,\text{g}\,\left(0\right)\,W\,D\,F^{\prime }},\mathrm{so}\,\frac{\partial E\,P\,e\,r}{\partial W\,D}=2\,F^{\prime }\,\frac{\partial e}{\partial W\,D}-1=\frac{1}{\text{g}\,\left(0\right)\,W\,D}-1$$

When the expected income is maximized, $\frac{\partial E\,P\,e\,r}{\partial W\,D}=0$, thus, the optimal compensation gap without fairness preference is: *WD* = $\frac{1}{\text{g}\,\left(0\right)}$. This shows that even under the pure self-interest hypothesis, the compensation gap has an interval effect on corporate performance. When the compensation gap *WD* is less than $\frac{1}{\text{g}\,\left(0\right)}$, the larger the compensation gap, the higher the corporate performance will be. When it is greater than $\frac{1}{\text{g}\,\left(0\right)}$, the larger the compensation gap, the lower the corporate performance will be. As a result, there is an inverted U-shaped relationship between the compensation gap and corporate performance, which is positive first and negative later.

### Tournament model based on fairness preference of agents

#### FS model

In this study, we choose the FS model of fairness preference theory model. The specific contents of the model are as follows:


$$U_{i}=x_{i}-\frac{\alpha_{i}}{n-1}\,\sum_{j\neq i}\max\left(x_{j}-x_{i},0\right)-\frac{\beta_{i}}{n-1}\,\sum_{j\neq i}\max\left(x_{i}-x_{j},0\right)$$

where *U*_*i*_ is the utility function of participant *i* and *x*_*i*_ is the income obtained by participant *i*. Both α and β are fairness preference intensity, and α is the disadvantage inequality aversion coefficient or jealousy intensity. The second term $\frac{\alpha_{i}}{n-1}\,\sum_{j\neq i}\max\left(x_{j}-x_{i},0\right)$ to the right of the equal sign represents the jealousy disutility of participant *i* affected by other (*n*−1) participants. β is the advantage inequality aversion coefficient or sympathy intensity. The third item $\frac{\beta_{i}}{n-1}\,\sum_{j\neq i}\max\left(x_{i}-x_{j},0\right)$ to the right of the equal sign represents the sympathy disutility of participant *i* affected by other (*n*−1) participants. There is a hypothesis α≥β, indicating that jealousy is often stronger than sympathy, and 0≤β < 1, indicating that although participants are sympathetic, they also like having a higher income than others. In particular, when the number of participants is two, the model is specifically expressed as:


$$U_{i}=x_{i}-\alpha_{i}\,\max\left(x_{j}-x_{i},0\right)-\beta_{i}\,\max\left(x_{i}-x_{j},0\right)$$

At this time, for a single participant, only one of the second or third terms to the right of the equal sign exists.

#### Tournament model based on fairness preference of agents

Introducing the FS model into the traditional tournament model, in the simple two-person model, it is assumed that the jealousy intensity and sympathy intensity between the two agents are the same, respectively. At this time, the agent will also compare with others’ incomes. The result of the comparison will have an effect on utility, which is specifically shown as follows:

When he(she) wins: *U^H^* = *W*_*H*_−β*WD*−*C*(*e*)

When he(she) fails: *U^L^* = *W*_*L*_−α*WD*−*C*(*e*)

Then, his(her) expected utility: *EU*_*i*_ = (1 + α−β)$P_{i}^{H}\,W\,D+W_{L}-\alpha \,W\,D-C\,\left(e_{i}\right)$

Dato et al. (2018) proved that behavioral symmetric equilibrium was reasonable even if participants had loss aversion based on expectation. Therefore, there is still:

When the expected utility is maximized:


(7) $$\frac{C^{\prime }}{F^{\prime }}=\text{g}\,\left(0\right)\,\left(1+\alpha -\beta \right)\,W\,D$$

Equation 7 shows that when *WD* is fixed, the increase in jealousy intensity α will improve the effort provision, while the increase in sympathy intensity β will reduce the effort provision. Since α > β in general, fairness preference under incentive compatibility constraints will improve the effort provision.

At the same time, under the participation constraint, there is:


(8) $$W_{L}+\frac{1}{2}\,\left(1-\alpha -\beta \right)\,W\,D-C\,\left(e\right)\geq U_{0}$$

where (α + β) is generally less than 1 (Grund and Sliwka, 2005), therefore, the positive relationship between effort provision *e* and compensation gap *WD* has not been changed. At the same time, Equation 8 also shows that under a given compensation gap *WD*, the greater the jealousy intensity α or sympathy intensity β is, the more the agent tends to reduce the effort provision *e* to meet the minimum reservation utility, that is, under the participation constraint, fairness preference will reduce the effort provision; for the principal, it is necessary to increase the compensation gap *WD* or compensation of loser *W_L_* to ensure that the agent can participate in the competition.

### Research hypotheses

#### The relationship between the compensation gap within the top management team and the corporate performance based on the tournament model considering fairness preference of agents

First, the tournament model based on fairness preference is analyzed in the same steps as the traditional tournament model:

Making the Equation 8 take the equal sign, we will get Equation 9:


(9) $$W_{L}+\frac{1}{2}\,\left(1-\alpha -\beta \right)\,W\,D-C\,\left(e\right)=U_{0}$$

The derivation of *WD* on both sides of Equation 9 can be obtained:


(10) $$\frac{1}{2}\,\left(1-\alpha -\beta \right)-C^{\prime }\,\frac{\partial e}{\partial W\,D}=0$$

From Equation 7, we know *C*′ = g(0)(1 + α−β)*WDF*′, then substitute it into Equation 10 to obtain:


$$\frac{1}{2}\,\left(1-\alpha -\beta \right)-\text{g}\,\left(0\right)\,\left(1+\alpha -\beta \right)\,W\,D\,F^{\prime }\,\frac{\partial e}{\partial W\,D}=0$$

So


(11) $$\frac{\partial e}{\partial W\,D}=\frac{1-\alpha -\beta }{2\,\text{g}\,\left(0\right)\,\left(1+\alpha -\beta \right)\,W\,D\,F^{\prime }}$$

Then, the principal’s expected income is *EPer* = 2*F*(*e*)−2*W*_*L*_−*WD*, and the derivation of *WD* is:


(12) $$\frac{\partial E\,P\,e\,r}{\partial W\,D}=2\,F^{\prime }\,\frac{\partial e}{\partial W\,D}-1=\frac{1-\alpha -\beta }{g\,\left(0\right)\,\left(1+\alpha -\beta \right)\,W\,D}-1$$

It can be seen that when the expected income is the largest, the value of the optimal compensation gap is *WD* = $\frac{1-\alpha -\beta }{\text{g}\,\left(0\right)\,\left(1+\alpha -\beta \right)}$. This shows that there is still an optimal compensation gap based on the fairness preference hypothesis, that is, the compensation gap still has an interval effect on corporate performance, which is consistent with the result in the traditional tournament model mentioned above. Based on this, we propose Hypothesis 1 as followings:

**Hypothesis 1:** There is an inverted U-shaped relationship between the TMT compensation gap and corporate performance.

**Hypothesis 1a:** Within the optimal compensation gap, the TMT compensation gap is positively related to corporate performance, and the larger the compensation gap, the higher the corporate performance will be.

**Hypothesis 1b:** When the optimal compensation gap is exceeded, the TMT compensation gap is negatively related to corporate performance, and the larger the compensation gap, the lower the corporate performance will be.

#### Moderating effect of fairness preference

Next, we discuss the moderating effect of fairness preference on the strength of the inverted U-shaped relationship between the compensation gap within the TMT and corporate performance. According to Equation 6, the derivative of expected income to compensation gap without fairness preference is $\frac{1}{\text{g}\,\left(0\right)\,W\,D}-1$, and according to Equation 12, the derivative of expected income to compensation gap with fairness preference is $\frac{1-\alpha -\beta }{\text{g}\,\left(0\right)\,\left(1+\alpha -\beta \right)\,W\,D}-1$. The analysis shows that $\frac{1-\alpha -\beta }{\text{g}\,\left(0\right)\,\left(1+\alpha -\beta \right)\,W\,D}-1<\frac{1}{\text{g}\,\left(0\right)\,W\,D}-1$, and this indicates that for the same level of compensation gap, due to the existence of fairness preference, its marginal contribution to corporate performance becomes smaller.

In order to investigate the influence of psychological intensity of fairness preference, we continue to take the derivation of fairness preference intensity with Equation 12, then obtain:


$$\frac{\partial^{2}E\,P\,e\,r}{\partial W\,D\,\partial \alpha }=\frac{-2+2\,\beta }{\text{g}\,\left(0\right)\,{\left(1+\alpha -\beta \right)}^{2}\,W\,D}<0$$

$$\frac{\partial^{2}E\,P\,e\,r}{\partial W\,D\,\partial \beta }=\frac{-2\,\alpha }{\text{g}\,\left(0\right)\,{\left(1+\alpha -\beta \right)}^{2}\,W\,D}<0$$

This means that for each level of compensation gap *WD*, the greater the jealousy intensity α or sympathy intensity β is, the lower the derivative of the principal’s expected income to the compensation gap is, that is, the marginal contribution of the compensation gap is lower.

In conclusion, the existence of fairness preference will reduce the marginal contribution of the compensation gap compared with that of pure self-interest, and the increase in fairness preference intensity will further reduce this marginal contribution. However, it is not certain whether this change includes the effect caused by the change of the extreme point. Therefore, Hypothesis 2 is proposed:

**Hypothesis 2:** Fairness preference moderates the correlation between the TMT compensation gap and corporate performance.

**Hypothesis 2a:** Within the optimal compensation gap, fairness preference will weaken the positive relationship between them.

**Hypothesis 2b:** When the optimal compensation gap is exceeded, fairness preference will strengthen the negative relationship between them.

## Methodology and results

### Sample and data collection

This study selects the panel data of A-share listed companies in Shenzhen and Shanghai stock markets from 2014 to 2020 as the research sample. The data are from the company research series database in the China Stock Market and Accounting Research (CSMAR) series research database. The industry classification is subject to the 2012 version of the China Securities Regulatory Commission. For the samples with missing executives’ annual compensation and educational background, a manual supplementary search is conducted on the webpage; the proportion of all kinds of personnel, including the proportion of technical staff, the proportion of undergraduate and above employees, and the proportion of management personnel, are all from the WIND database.

First, based on the original samples, this study preliminarily carried out the following processing: (1) nonfinancial industries and non-ST enterprises during the sample observation period were selected to make the samples more robust and eliminate the influence of outliers; (2) the enterprise samples with missing compensation data were excluded; and (3) enterprises with fewer than 100 employees were excluded to make the samples more representative.

Second, because the compensation gap within the TMT is a core variable of this study, so it is particularly important to identify the members of the TMT. Through the analysis of the theoretical model, employees holding the positions such as general manager, deputy general manager, board secretary, and so on, were selected as the members of the TMT, which does not include non-part-time directors and supervisors. On this basis, further processing was done as follows: (1) in view of the definition of a team, samples with less than 2 senior executives were excluded; (2) referring to the practice of Chen and Zhang (2010), the samples whose highest annual compensation is non-CEO in the TMT were excluded. So far, the sample data of 733 corporate executives have been obtained.

Finally, referring to the general data processing methods of empirical study, we carried out winsorizing of 0.01 up and down for continuous variables. Additionally, the unbalanced panel data of 3,093 effective observations of 733 A-share listed companies from 2014 to 2020 were obtained.

### Model construction

#### The inverted U-shaped relationship between the compensation gap within the top management team and the corporate performance

For the test of inverted U-shaped relationship, the squared term of the explanatory variable, including the one-degree term of the explanatory variable and other control variables, must be added to the compact model. After the regression, the analysis and judgment are made according to the significance and symbol of the one-degree term and the squared term in the results. Therefore, referring to the test model developed by Chen and Zhang (2010), we first established Model 1 to verify the relationship between the compensation gap *WD* within the TMT and the corporate performance *PER*:


$$\text{Model 1}:P\,E\,R_{i,t}=\alpha_{0}+\alpha_{1}\,W\,D_{i,t}+\alpha_{2}\,W\,D_{i,t}^{2}+\alpha_{3}\,C_{i,t}+\varepsilon_{i,t}$$

For the explained variable corporate performance *PER*, according to Gao and Lu (2015), the market performance with less incentive effect on executives was not used, and the three indicators of *ROA* (Lin et al., 2003), *EPS* (Zhang, 2007), and *ROE* (Lu H., 2007; Li et al., 2014) were used as the explained variable of the research subject. For the explanatory variable, the compensation gap *WD* within the TMT, referring to the research of Chen and Zhang (2010) and Yang and Wang (2014), two absolute indicators were used to measure. The first was *WDl*, which was used to measure the difference between the highest and the lowest compensation in the team, and the second was *WDa*, which was used to measure the difference between the highest and the average compensation in the team. So far, six groups of specific models as followings have been included:


$$\text{Model 1-}\,1:R\,O\,A_{i,t}=\alpha_{0}+\alpha_{1}\,W\,D\,l_{i,t}+\alpha_{2}\,W\,D\,l\,s\,q_{i,t}+\alpha_{3}\,C_{i,t}+\varepsilon_{i,t}$$

$$\text{Model 1-}\,2:R\,O\,A_{i,t}=\alpha_{0}+\alpha_{1}\,W\,D\,a_{i,t}+\alpha_{2}\,W\,D\,a\,s\,q_{i,t}+\alpha_{3}\,C_{i,t}+\varepsilon_{i,t}$$

$$\text{Model 1-}\,3:E\,P\,S_{i,t}=\alpha_{0}+\alpha_{1}\,W\,D\,l_{i,t}+\alpha_{2}\,W\,D\,l\,s\,q_{i,t}+\alpha_{3}\,C_{i,t}+\varepsilon_{i,t}$$

$$\text{Model 1-}\,4:E\,P\,S_{i,t}=\alpha_{0}+\alpha_{1}\,W\,D\,a_{i,t}+\alpha_{2}\,W\,D\,a\,s\,q_{i,t}+\alpha_{3}\,C_{i,t}+\varepsilon_{i,t}$$

$$\text{Model 1-}\,5:R\,O\,E_{i,t}=\alpha_{0}+\alpha_{1}\,W\,D\,l_{i,t}+\alpha_{2}\,W\,D\,l\,s\,q_{i,t}+\alpha_{3}\,C_{i,t}+\varepsilon_{i,t}$$

$$\text{Model 1-}\,6:R\,O\,E_{i,t}=\alpha_{0}+\alpha_{1}\,W\,D\,a_{i,t}+\alpha_{2}\,W\,D\,a\,s\,q_{i,t}+\alpha_{3}\,C_{i,t}+\varepsilon_{i,t}$$

#### Measurement of fairness preference intensity

Yan and Jin (2014) believed that the degree of compensation inequity and educational background could reflect the fairness preference intensity of senior executives in state-owned enterprises, so they set up these two indicators as the substitute variables of fairness preference, in which the degree of compensation inequity was obtained by using modeling regression to obtain the residual according to the research of Cowherd and Levine (1992). Based on the practices of Yan and Jin (2014) and Cowherd and Levine (1992), this article first obtains the numerical *DCOM* of the compensation gap between senior executives and the industry and takes it as the explained variable to investigate the degree of compensation inequity that can be explained by the human capital characteristics of senior executives such as gender, age, tenure, educational background, professional title, and so on, as well as the characteristics of the enterprise, time and industry, that is, the model residual. The absolute value is taken to represent the fairness preference intensity. However, after analysis, as the role brought by the external environment, the degree of external compensation inequity can only represent the strength of senior executives’ fairness preference and cannot directly represent whether the specific preference of senior executives is jealousy or sympathy. Therefore, it is only to obtain the absolute value of the residual and does not distinguish the degree of inequity of advantages and disadvantages as Yan and Jin (2014). Based on this, Model 2 is established:

Model 2:


$$D\,C\,O\,M_{i,t}=\beta_{0}+\beta_{1}\,G\,E\,N_{i,t}+\beta_{2}\,A\,G\,E_{i,t}+\beta_{3}\,T\,E\,N_{i,t}+\beta_{4}\,B\,G_{i,t}$$

$$+\beta_{5}\,P\,R\,F_{i,t}+\beta_{6}\,L\,N\,N_{i,t}+\beta_{7}\,R\,O\,A_{i,t}+\beta_{8}\,Y\,E\,A\,R$$

$$+\beta_{9}\,I\,N\,D\,U\,S+\varepsilon_{i,t}$$

#### The role of fairness preference

In order to verify the moderating effect of fairness preference on the relationship between the compensation gap within the TMT and the corporate performance and its impact on the optimal compensation gap, we need to add the index of fairness preference *Z*, the interaction between fairness preference and the one-degree term of explanatory variable *WD*, and the interaction between the fairness preference and the squared term of *WD* into Model 1, to establish Model 3:

Model 3:


$$P\,E\,R_{i,t}=\lambda_{0}+\lambda_{1}\,W\,D_{i,t}+\lambda_{2}\,W\,D_{i,t}^{2}+\lambda_{3}\,Z_{i,t}\cdot W\,D_{i,t}$$

$$+\lambda_{4}\,Z_{i,t}\cdot W\,D_{i,t}^{2}+\lambda_{5}\,Z_{i,t}+\lambda_{6}\,C_{i,t}+\varepsilon_{i,t}$$

According to Model 2, in the main part of the study, the degree of compensation inequity *F* is added to Model 3 as a substitute variable of fairness preference *Z*, and six groups of models including three explained variables and two explanatory variables are also obtained, which are as followings:

Model 3-1:


$$R\,O\,A_{i,t}=\alpha_{0}+\alpha_{1}\,W\,D\,l_{i,t}+\alpha_{2}\,W\,D\,l\,s\,q_{i,t}+\alpha_{3}\,F_{i,t}\cdot W\,D\,l_{i,t}$$

$$+\alpha_{4}\,F_{i,t}\cdot W\,D\,l\,s\,q_{i,t}+\alpha_{5}\,F_{i,t}+\alpha_{6}\,C_{i,t}+\varepsilon_{i,t}$$

Model 3-2:


$$R\,O\,A_{i,t}=\alpha_{0}+\alpha_{1}\,W\,D\,a_{i,t}+\alpha_{2}\,W\,D\,a\,s\,q_{i,t}+\alpha_{3}\,F_{i,t}\cdot W\,D\,a_{i,t}$$

$$+\alpha_{4}\,F_{i,t}\cdot W\,D\,a\,s\,q_{i,t}+\alpha_{5}\,F_{i,t}+\alpha_{6}\,C_{i,t}+\varepsilon_{i,t}$$

Model 3-3:


$$E\,P\,S_{i,t}=\alpha_{0}+\alpha_{1}\,W\,D\,l_{i,t}+\alpha_{2}\,W\,D\,l\,s\,q_{i,t}+\alpha_{3}\,F_{i,t}\cdot W\,D\,l_{i,t}$$

$$+\alpha_{4}\,F_{i,t}\cdot W\,D\,l\,s\,q_{i,t}+\alpha_{5}\,F_{i,t}+\alpha_{6}\,C_{i,t}+\varepsilon_{i,t}$$

Model 3-4:


$$E\,P\,S_{i,t}=\alpha_{0}+\alpha_{1}\,W\,D\,a_{i,t}+\alpha_{2}\,W\,D\,a\,s\,q_{i,t}+\alpha_{3}\,F_{i,t}\cdot W\,D\,a_{i,t}$$

$$+\alpha_{4}\,F_{i,t}\cdot W\,D\,a\,s\,q_{i,t}+\alpha_{5}\,F_{i,t}+\alpha_{6}\,C_{i,t}+\varepsilon_{i,t}$$

Model 3-5:


$$R\,O\,E_{i,t}=\alpha_{0}+\alpha_{1}\,W\,D\,l_{i,t}+\alpha_{2}\,W\,D\,l\,s\,q_{i,t}+\alpha_{3}\,F_{i,t}\cdot W\,D\,l_{i,t}$$

$$+\alpha_{4}\,F_{i,t}\cdot W\,D\,l\,s\,q_{i,t}+\alpha_{5}\,F_{i,t}+\alpha_{6}\,C_{i,t}+\varepsilon_{i,t}$$

Model 3-6:


$$R\,O\,E_{i,t}=\alpha_{0}+\alpha_{1}\,W\,D\,a_{i,t}+\alpha_{2}\,W\,D\,a\,s\,q_{i,t}+\alpha_{3}\,F_{i,t}\cdot W\,D\,a_{i,t}$$

$$+\alpha_{4}\,F_{i,t}\cdot W\,D\,a\,s\,q_{i,t}+\alpha_{5}\,F_{i,t}+\alpha_{6}\,C_{i,t}+\varepsilon_{i,t}$$

### Variables definition

The definition of all variables involved in this study is shown in Table 1.

**TABLE 1:** Variable connotation.

Variables	Meaning	Definition
**Models 1, 3**		
*PER*	Corporate performance	Explained variable
*ROA*	Return on assets (%)	Net profit divided by average total assets
*EPS*	Basic earnings per share	The current period net profit attributable to common stockholders divided by the weighted average number of ordinary shares outstanding in the current period
*ROE*	Return on equity (%)	Net profit divided by balance of shareholders’ equity
*WD*	Compensation gap within the TMT	Explanatory variable
*WDl*	Absolute index 1 (10,000)	CEO compensation minus team minimum compensation
*WDlsq*	Squared term 1	(CEO compensation minus team minimum compensation)^2^
*WDa*	Absolute index 2 (10,000)	CEO compensation minus average team compensation
*WDasq*	Squared term 2	(CEO compensation minus average team compensation)^2^
*Z*	Fairness preference	Moderating variable
*F*	The degree of external compensation inequity	Absolute value of the residual for Model 2 regression
*BG*	Educational background	Average education background of the TMT
*C*		Control variables
*LNN*	Firm size	Ln (number of people in the enterprise)
*POT*	The proportion of technical staff (%)	Number of technical staff divided by number of employees
*POM*	The proportion of management personnel (%)	Number of management personnel divided by number of employees
*PUT*	The proportion of undergraduate and above employees (%)	Number of undergraduate and above employees divided by number of employees
*PSS*	Proportion of state-owned shares (%)	The number of state-owned shares divided by the total number of shares
*TOP10*	Ownership concentration (%)	Proportion of top 10 circulating shares
*BOD*	Board size	Number of board members
*IDP*	Proportion of independent directors (%)	Number of independent directors divided by board size
*DUAL*	CEO duality	Dummy variable, which takes 1 when the CEO and the chairman of the board are the same person, otherwise takes 0
*YEAR*	Year	Dummy variable
**Model 2**		
*DCOM*	The compensation gap with the industry (10,000)	The average compensation of the top three executives minus the average compensation of the top three executives in the industry
*GEN*	Gender (%)	The number of male executives divided by the number of people in the TMT
*AGE*	Age	Average age of the TMT
*TEN*	Tenure	Average tenure of the TMT
*BG*	Educational background	Average education background of the TMT
*PRF*	Professional title (%)	The number of executives with professional titles divided by the number of people in the TMT
*LNN*	Same as Models 1, 3	—
*ROA*	Same as Models 1, 3	—
*INDUS*	Industry	Dummy variable
*YEAR*	Year	Dummy variable

In Model 1 and Model 3:

1. Explained variable *PER*: As mentioned in the model construction, corporate performance is measured by three indicators: *ROA, EPS*, and *ROE*.
2. Explanatory variable *WD*: As mentioned in the model construction, two absolute gap indicators *WDl* and *WDa* are used to measure the compensation gap within the TMT, and *WDlsq* and *WDasq* represent the squared terms of *WDl* and *WDa*, respectively, to test the inverted U-shaped relationship between *PER* and *WD*.
3. Moderating variable *Z*: As mentioned in the model construction, the fairness preference is measured by two indicators, namely, the degree of external compensation inequity *F* and educational background *BG*. Among them, the former indicator *F* is used for the subject regression, which is obtained by taking the absolute value of the residual term obtained by the regression of Model 2; the latter indicator *BG* is used for the robustness test, which is obtained by taking the average of the education background of the TMT. The educational background of each member of the team is as follows: (1) for below junior college and other educational backgrounds, (2) for junior college, (3) for undergraduate, (4) for postgraduate, and (5) for doctoral students and postdoctoral. For the executives who cannot find their educational background in all ways, then it is classified as other, numbered as 1. The larger the BG value is, the higher the overall educational level of the TMT is.
4. Control variables C: 10 control variables are selected in this study, as shown in Table 1.

In Model 2:

1. Explained variable *DCOM*: As mentioned in the model construction, it is calculated from the difference between the average compensation of the top three executives in the enterprise and the average compensation of the top three executives in the industry.
2. Explanatory variables: Nine explanatory variables are used. The first five variables gender *GEN*, age *AGE*, tenure *TEN*, educational background BG, and professional title PRF are responsible for explaining the industry compensation gap caused by the human capital characteristics of the TMT, and firm size *LNN* and return on assets *ROA* explain *DCOM* from the enterprise management level, while also controlling the industry and time. Among them, the *TEN* variable of tenure is calculated by comparing the job start date and job end date of the non-director or supervisor positions held by senior executives with the sample observation time, and for senior executives who hold several positions concurrently, we select the longest term. Industry *INDUS* dummy variable includes 16 industry categories.

### Empirical test results and analysis

In this study, EXCEL and STATA software are used for research data processing and regression of the model. The results are as follows:

#### Descriptive statistics

Table 2 lists the descriptive statistical results of the main variables of each model in this study. It can be seen that the maximum value of *WDl* is 5,900,000, the minimum value is 40,400; the maximum value of *WDa* is 3,821,820, and the minimum value is 24,500, with a difference of about 146 times and 155 times, respectively, which shows the great difference in the compensation gap within the TMT of different enterprises in China. In addition, from an annual perspective, except for the changes in the minimum values of *WDl* and *WDa* in 2019, the average values of *WDl* and *WDa* show an increasing trend year by year when their maximum and minimum values remain unchanged, indicating that the compensation gap within the TMT of China’s nonfinancial enterprises is expanding year by year.

**TABLE 2:** Descriptive statistics of variables.

Variables	Mean	SD	Min	Max
**Models 1, 3**				
*ROA*	4.477	5.171	−16.415	18.563
*ROE*	7.183	9.4	−41.142	30.157
*EPS*	0.384	0.481	−1.04	2.29
*WDl*	71.767	95.183	4.04	590
*WDa*	42.122	61.12	2.45	382.182
*LNN*	7.72	1.148	5.226	10.959
*POT*	20.336	16.708	0	82.19
*POM*	2.509	5.602	0	28.65
*PUT*	24.965	19.716	0	86.393
*PSS*	1.829	6.951	0	45.735
*TOP10*	41.803	20.487	1.552	87.563
*BOD*	8.486	1.545	5	14
*IDP*	37.427	5.283	33.333	57.143
*DUAL*	0.29	0.454	0	1
*F*	44.35985	59.40454	0.0154	495.9888
*BG*	3.221	0.525	1.833	4.333
**Model 2**				
*DCOM*	2.122	76.186	−106.706	430.516
*GEN*	82.994	16.512	33.333	100
*AGE*	47.944	3.528	39	56.25
*TEN*	5.103	2.21	0.977	11.538
*BG*	3.221	0.525	1.833	4.333
*PRF*	45.849	31.913	0	100
**WDl**				
2014	52.7	68.67	4.04	590
2015	57.67	78.93	4.04	590
2016	65.75	87.79	4.04	590
2017	72.37	91.32	4.04	590
2018	83.56	105.2	4.04	590
2019	129.1	138.5	5.84	590
2020	146.2	153.3	4.04	590
**WDa**				
2014	31.04	44.04	2.45	382.182
2015	34.84	52.86	2.45	382.182
2016	38.92	56.77	2.45	382.182
2017	42.47	58.71	2.45	382.182
2018	49.31	67.99	2.45	382.182
2019	70.92	87.11	2.96	382.182
2020	83.71	100.6	2.45	382.182

#### Multicollinearity test

In order to avoid the decline of the single explanatory power of model parameter estimation caused by multicollinearity, the multicollinearity test was carried out on the explanatory variable of the samples for Model 1, and the variance inflation factor (VIF) under the two *WD* indicators was obtained, as shown in Table 3.

**TABLE 3:** Multicollinearity test.

Variables	VIF (compact)	VIF	Variables	VIF (compact)	VIF
*WDl*	1.27	7.97	*WDa*	1.22	8.09
*WDlsq*		7.55	*WDasq*		7.76
*LNN*	1.37	1.37	*LNN*	1.35	1.35
*POT*	1.78	1.78	*POT*	1.77	1.78
*POM*	1.25	1.25	*POM*	1.25	1.25
*PUT*	1.91	1.91	*PUT*	1.9	1.9
*PSS*	1.08	1.08	*PSS*	1.08	1.08
*TOP10*	1.21	1.21	*TOP10*	1.21	1.21
*BOD*	1.66	1.66	*BOD*	1.66	1.66
*IDP*	1.55	1.55	*IDP*	1.55	1.55
*DUAL*	1.08	1.08	*DUAL*	1.08	1.08
2015	1.66	1.66	2015	1.66	1.66
2016	1.7	1.7	2016	1.7	1.7
2017	1.72	1.72	2017	1.72	1.72
2018	1.75	1.76	2018	1.75	1.75
2019	1.41	1.42	2019	1.4	1.41
2020	1.22	1.22	2020	1.21	1.21
VIF mean	1.48	2.23	VIF mean	1.47	2.24

First, as a comparison, the VIF of all variables in the compact Model 1 (excluding the squared term of *WD*) does not exceed 2, and the average VIF is 1.48 and 1.47, respectively, which shows that the correlation between variables in compact Model 1 is weak, so there is no need to worry about the multicollinearity problems of the compact model. Second, the average values of VIF under the test of the two groups of complete Model 1 are 2.23 and 2.24, respectively, both of which do not exceed 3. Among them, the VIF of the control variables is less than 2, and the VIF of the one-degree term and the squared term of the explanatory variable *WD* is larger but does not exceed 10. Therefore, it can be considered that the correlation between the variables in Model 1 is not strong, and multicollinearity is not a serious problem.

#### Model test results and analysis

In this study, the two-way fixed effects model including individual effects and time effects under cluster robust standard errors is selected for regression.

##### Scatter plot analysis

**FIGURE 1:**
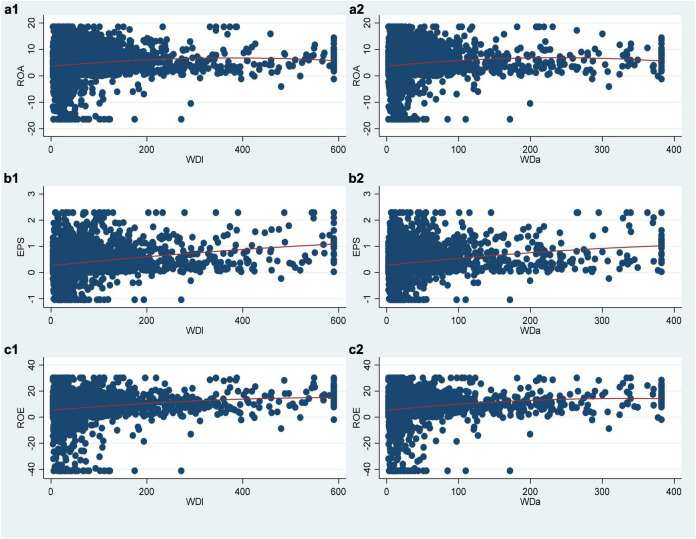
Sample scatter plot. **(a1–c2)** Represent the relationship between different variables.

Figure 1 shows the scatter plot and qfit fitting between the performance indicators of each enterprise and the compensation gap indicators within each TMT.

First, it can be seen from the six scatter plots that the sample observations are widely distributed within the range of explanatory variable *WD*, especially when *WDl* is less than 2,000,000 and *WDa* is less than 1,000,000. Second, qfit fitting shows that in the six figures, except (b1) and (b2), namely, when the corporate performance takes the *EPS* index, the fitting line does not have obvious bending, and the other four figures all show a more obvious inverted U-shaped fitting shape. Therefore, through the observation of the scatter plots, it can be preliminarily judged that there is an inverted U-shaped relationship between the compensation gap *WD* within the TMT and the corporate performance *PER*, and most of the samples are in an upward stage of the inverted U-shaped.

##### Results analysis without considering fairness preference of agents

The six groups of models in Model 1 are regressed, respectively, and the results are shown in Table 4.

**TABLE 4:** Model 1 regression results.

Variables	Model 1-1 (*ROA*)	Model 1-2 (*EPS*)	Model 1-3 (*ROE*)	Variables	Model 1-4 (*ROA*)	Model 1-5 (*EPS*)	Model 1-6 (*ROE*)
*WDl*	0.0137*** (0.00512)	0.00119*** (0.000419)	0.0233** (0.0107)	*WDa*	0.0233** (0.00914)	0.00220*** (0.000723)	0.0420** (0.0194)
*WDlsq*	−1.67e-05** (7.72e-06)	−1.17e-06 (7.60e-07)	−2.64e-05* (1.51e-05)	*WDasq*	−4.38e-05** (2.13e-05)	−3.79e-06* (1.96e-06)	−7.68e-05* (4.28e-05)
*LNN*	0.302 (0.435)	0.0475* (0.0265)	1.209 (1.007)	*LNN*	0.299 (0.434)	0.0470* (0.0265)	1.199 (1.003)
*POT*	0.0200 (0.0155)	0.00209* (0.00116)	0.0396 (0.0298)	*POT*	0.0204 (0.0154)	0.00210* (0.00115)	0.0400 (0.0296)
*POM*	0.0370** (0.0184)	0.00165 (0.00154)	0.0504 (0.0377)	*POM*	0.0366** (0.0182)	0.00161 (0.00153)	0.0495 (0.0374)
*PUT*	0.0180 (0.0182)	0.000576 (0.00113)	0.0270 (0.0334)	*PUT*	0.0178 (0.0182)	0.000544 (0.00112)	0.0266 (0.0333)
*PSS*	−0.0310* (0.0176)	−0.00181 (0.00132)	−0.0862** (0.0365)	*PSS*	−0.0309* (0.0176)	−0.00182 (0.00132)	−0.0863** (0.0366)
*TOP10*	−0.0339*** (0.00912)	−0.00351*** (0.000801)	−0.0605*** (0.0174)	*TOP10*	−0.0338*** (0.00912)	−0.00351*** (0.000801)	−0.0604*** (0.0174)
*BOD*	0.121 (0.179)	0.0110 (0.0162)	0.416 (0.368)	*BOD*	0.117 (0.178)	0.0108 (0.0161)	0.410 (0.368)
*IDP*	0.0353 (0.0388)	0.00192 (0.00293)	0.0895 (0.0768)	*IDP*	0.0340 (0.0390)	0.00180 (0.00293)	0.0871 (0.0769)
*DUAL*	0.342 (0.520)	0.0371 (0.0348)	0.835 (1.106)	*DUAL*	0.314 (0.519)	0.0334 (0.0345)	0.774 (1.100)
Constant	−0.262 (4.160)	−0.0975 (0.298)	−8.763 (8.837)	Constant	−0.158 (4.167)	−0.0899 (0.298)	−8.578 (8.836)
Individual fixed effects	Yes	Yes	Yes	Individual fixed effects	Yes	Yes	Yes
Time fixed effects	Yes	Yes	Yes	Time fixed effects	Yes	Yes	Yes
Observation	3,093	3,093	3,093	Observation	3,093	3,093	3,093
*R* ^2^	0.024	0.041	0.023	*R* ^2^	0.025	0.042	0.024

The symbols *, **, and *** denote significance at the 10, 5, and 1% significance levels, respectively.

Robust standard errors are presented in parentheses.

##### Inverted U-shaped relationship analysis

The purpose of Model 1 is to test whether there is an inverted U-shaped relationship between the explained variable *PER* and the explanatory variable *WD*. The key to the test is that the coefficient of the one-degree term of *WD* in the model regression is significantly positive, and the coefficient of the squared term is significantly negative.

According to Table 4, we first investigate the significance and direction of the coefficients of the one-degree term (*WDl* and *WDa*) of the explanatory variable *WD*. It can be seen that Model 1-1, Model 1-2, and Model 1-5 are significant at the level of 1%, Model 1-3, Model 1-4, and Model 1-6 are significant at the level of 5%, and the one-degree term coefficients of the six groups of models are all positive. Second, the significance and direction of the coefficients of the squared term *WD*^2^ (*WDlsq* and *WDasq*) of *WD* are investigated. It is found that Model 1-1 and Model 1-4 are significant at the level of 5%, Model 1-3, Model 1-5, and Model 1-6 are significant at the level of 10%, Model 1-2 is not significant, and the squared term coefficients of the six groups of models are all negative. It can be considered that there is a nonlinear relationship between the compensation gap *WD* within the TMT and the corporate performance *PER*. At the same time, combined with the result that the one-degree term coefficient is positive, and the squared term coefficient is negative, it can be determined that the nonlinear relationship between the compensation gap *WD* within the TMT and the corporate performance PER is an inverted U-shape with positive first and then negative. That is, when *WD* is within the optimal compensation gap, the larger the *WD* is, the better the corporate performance PER. When the *WD* exceeds the optimal compensation gap, the larger the *WD* is, the worse the corporate performance PER. Hypothesis 1, Hypothesis 1a, and Hypothesis 1b are supported.

##### Marginal effects plot

**FIGURE 2:**
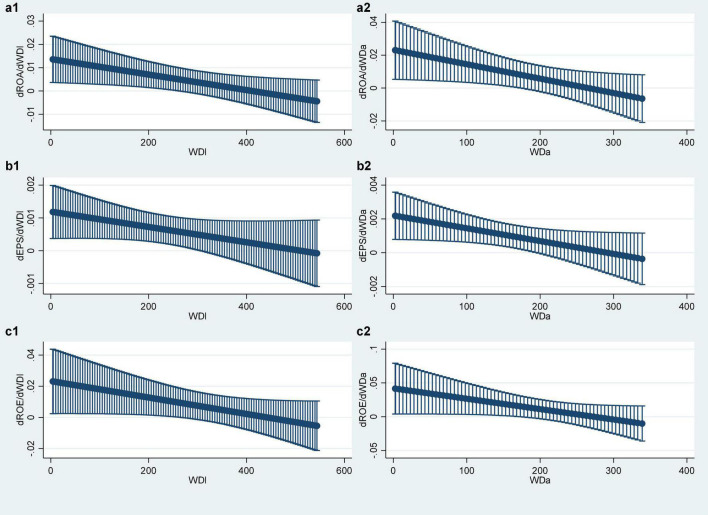
Marginal contribution of WD. **(a1–c2)** Represent the relationship between different variables.

In order to more intuitively show the marginal contribution of the compensation gap *WD* within the TMT to the corporate performance PER, this study draws the *WD* marginal effects plot, as shown in Figure 2:

It can be seen from Figure 2 that among the six groups of models, there is a phenomenon that the marginal contribution value of the compensation gap *WD* within the TMT changes from positive to negative and gradually decreases. This shows that the corporate performance, as described in the above regression results analysis, will first increase and then decline with the increase in the compensation gap within the TMT.

##### Results analysis considering fairness preference of agents

The absolute value of the residual obtained by the regression of Model 2 is substituted into Model 3 for regression. The results of the six groups of models are shown in Table 5:

**TABLE 5:** Model 3 regression results.

Variables	Model 3-1 (*ROA*)	Model 3-2 (*EPS*)	Model 3-3 (*ROE*)	Variables	Model 3-4 (*ROA*)	Model 3-5 (*EPS*)	Model 3-6 (*ROE*)
*WDl*	0.0132*** (0.00425)	0.00108*** (0.000356)	0.0220** (0.00879)	*WDa*	0.0231*** (0.00787)	0.00197*** (0.000643)	0.0406** (0.0164)
*WDlsq*	−2.67e-05* (1.45e-05)	−2.56e-06* (1.50e-06)	−4.79e-05* (2.88e-05)	*WDasq*	−6.67e-05** (3.36e-05)	−6.72e-06** (3.37e-06)	−0.000128* (6.70e-05)
*F*	0.0260*** (0.00765)	0.00152** (0.000595)	0.0556*** (0.0171)	*F*	0.0239*** (0.00717)	0.00135** (0.000559)	0.0511*** (0.0160)
*F* × *WDl*	−0.000175*** (4.08e-05)	−5.82e-06 (3.97e-06)	−0.000323*** (8.47e-05)	*F* × *WDa*	−0.000262*** (6.18e-05)	−7.24e-06 (5.92e-06)	−0.000478*** (0.000133)
*F* × *WDlsq*	2.74e-07*** (6.09e-08)	1.07e-08* (6.33e-09)	4.99e-07*** (1.24e-07)	*F* × *WDasq*	6.32e-07*** (1.49e-07)	2.14e-08 (1.53e-08)	1.15e-06*** (3.13e-07)
*LNN*	0.206 (0.419)	0.0429* (0.0259)	1.019 (0.980)	*LNN*	0.229 (0.422)	0.0437* (0.0260)	1.058 (0.979)
*POT*	0.0227 (0.0154)	0.00222* (0.00116)	0.0450 (0.0297)	*POT*	0.0223 (0.0154)	0.00221* (0.00116)	0.0439 (0.0299)
*POM*	0.0378** (0.0189)	0.00153 (0.00156)	0.0505 (0.0387)	*POM*	0.0376** (0.0188)	0.00148 (0.00155)	0.0500 (0.0385)
*PUT*	0.0146 (0.0180)	0.000415 (0.00115)	0.0203 (0.0331)	*PUT*	0.0145 (0.0180)	0.000412 (0.00114)	0.0202 (0.0331)
*PSS*	−0.0300* (0.0172)	−0.00178 (0.00133)	−0.0843** (0.0357)	*PSS*	−0.0304* (0.0173)	−0.00179 (0.00132)	−0.0851** (0.0360)
*TOP10*	−0.0323*** (0.00907)	−0.00343*** (0.000803)	−0.0573*** (0.0172)	*TOP10*	−0.0324*** (0.00908)	−0.00344*** (0.000801)	−0.0576*** (0.0172)
*BOD*	0.126 (0.181)	0.0110 (0.0164)	0.427 (0.373)	*BOD*	0.125 (0.180)	0.0109 (0.0164)	0.423 (0.373)
*IDP*	0.0327 (0.0386)	0.00174 (0.00293)	0.0840 (0.0765)	*IDP*	0.0312 (0.0388)	0.00168 (0.00293)	0.0816 (0.0766)
*DUAL*	0.340 (0.511)	0.0374 (0.0342)	0.842 (1.087)	*DUAL*	0.315 (0.512)	0.0345 (0.0341)	0.792 (1.082)
Constant	1.940 (4.044)	0.0501 (0.295)	−4.627 (8.591)	Constant	1.832 (4.069)	0.0474 (0.297)	−4.789 (8.584)
Individual fixed effects	Yes	Yes	Yes	Individual fixed effects	Yes	Yes	Yes
Time fixed effects	Yes	Yes	Yes	Time fixed effects	Yes	Yes	Yes
Observation	3,093	3,093	3,093	Observation	3,093	3,093	3,093
*R* ^2^	0.036	0.047	0.035	*R* ^2^	0.035	0.048	0.035

The symbols *, **, and *** denote significance at the 10, 5, and 1% significance levels, respectively.

Robust standard errors are presented in parentheses.

**FIGURE 3:**
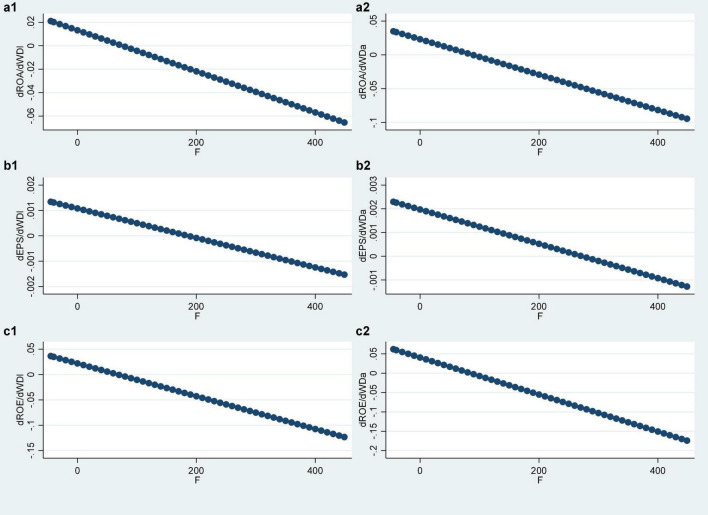
Moderating effects of fairness preference. **(a1–c2)** Represent the relationship between different variables.

The moderating effects plot of fairness preference of the six groups of models in Model 3 is shown in Figure 3. The horizontal coordinate is the intensity of fairness preference, and the vertical coordinate is the average marginal contribution of the compensation gap *WD* within the TMT to the corporate performance *PER*. The average marginal contribution of *WD* in the six figures all show a trend of decreasing with the increase in the intensity of fairness preference, that is, the stronger the fairness preference is, the smaller the derivative of the corporate performance PER to the compensation gap *WD* within the TMT. That confirms the preliminary results in the derivation of the Hypothesis 2 theoretical model.

Observing the regression results in Table 5, first of all, the quadratic interaction terms (*F* × *WDlsq, F* × *WDasq*) in Model 3-1, Model 3-3, Model 3-4, and Model 3-6 are all significant at the level of 1%, Model 3-2 is significant at the level of 10%, and Model 3-5 is not significant. It can be considered that fairness preference *Z* (*F*) has a significant impact on the marginal contribution of the compensation gap *WD* within the TMT. Second, in the six groups of models, the symbols of the coefficients of the quadratic interaction terms (*F* × *WDlsq, F* × *WDasq*) are all positive, which indicates that the stronger the fairness preference is, the smaller the absolute value of the marginal contribution of the compensation gap *WD* within the TMT is. In conclusion, fairness preference can moderate the relationship between the compensation gap within the TMT and corporate performance, which supports Hypothesis 2. At the same time, this moderating effect shows as a weakening effect. That is, on the left side of the optimal compensation gap, fairness preference will weaken the positive relationship between the two, while on the right side of the optimal compensation gap, it will weaken the negative relationship between the two, so Hypothesis 2a is supported, but Hypothesis 2b is not supported.

##### Robustness tests

The robustness test of this study first reports the compact model of Model 1 to verify whether the direction and significance of the coefficients of the complete model are stable. Then, for Model 3, the index of fairness preference is replaced to compare and test the original moderating model.

##### Compact Model 1 regression

In order to test whether the results of Model 1 are robust, Table 6 lists six groups of compact Models in which Model 1 only contains the one-degree term of the explanatory variable. It can be seen from Table 6 that the regression coefficient of *WDl* in compact Model 1-2 is significantly positive at the level of 1%, the regression coefficients of *WDl* and *WDa* in compact Model 1-1, compact Model 1-3, and compact Model 1-5 are all significantly positive at the level of 5%, and the regression coefficients of WDa in compact Model 1-4 and compact Model 1-6 are significantly positive at the level of 10%. It indicates that when using the linear model, there is a significant positive relationship between the compensation gap within the TMT and corporate performance. The larger the compensation gap within the TMT, the higher the corporate performance. This result does not change the coefficient significance and symbolic direction of the one-degree term of the explanatory variable in Table 4, indicating that the regression results of the complete Model 1 with the one degree and squared terms of the compensation gap within the TMT listed in Table 4 are relatively robust.

**TABLE 6:** Compact Model 1 containing only the one-degree term of WD.

Variables	Compact Model 1-1 (*ROA*)	Compact Model 1-2 (*EPS*)	Compact Model 1-3 (*ROE*)	Variables	Compact Model 1-4 (*ROA*)	Compact Model 1-5 (*EPS*)	Compact Model 1-6 (*ROE*)
*WDl*	0.00527** (0.00261)	0.000599*** (0.000220)	0.00993** (0.00494)	*WDa*	0.00834* (0.00426)	0.000913** (0.000354)	0.0158* (0.00812)
*LNN*	0.327 (0.432)	0.0492* (0.0264)	1.249 (1.005)	*LNN*	0.325 (0.432)	0.0492* (0.0265)	1.244 (1.004)
*POT*	0.0218 (0.0155)	0.00221* (0.00114)	0.0423 (0.0294)	*POT*	0.0220 (0.0154)	0.00224** (0.00114)	0.0427 (0.0293)
*POM*	0.0378** (0.0182)	0.00171 (0.00153)	0.0516 (0.0375)	*POM*	0.0377** (0.0181)	0.00170 (0.00152)	0.0514 (0.0373)
*PUT*	0.0190 (0.0182)	0.000646 (0.00112)	0.0286 (0.0332)	*PUT*	0.0188 (0.0181)	0.000634 (0.00112)	0.0284 (0.0331)
*PSS*	−0.0300* (0.0176)	−0.00174 (0.00133)	−0.0847** (0.0365)	*PSS*	−0.0302* (0.0176)	−0.00176 (0.00133)	−0.0850** (0.0365)
*TOP10*	−0.0338*** (0.00913)	−0.00351*** (0.000802)	−0.0604*** (0.0174)	*TOP10*	−0.0338*** (0.00913)	−0.00351*** (0.000804)	−0.0604*** (0.0174)
*BOD*	0.114 (0.178)	0.0105 (0.0161)	0.405 (0.366)	*BOD*	0.111 (0.177)	0.0104 (0.0160)	0.400 (0.365)
*IDP*	0.0349 (0.0388)	0.00190 (0.00292)	0.0889 (0.0767)	*IDP*	0.0337 (0.0388)	0.00178 (0.00292)	0.0866 (0.0768)
*DUAL*	0.401 (0.522)	0.0413 (0.0351)	0.930 (1.107)	*DUAL*	0.389 (0.521)	0.0400 (0.0350)	0.906 (1.105)
Constant	−0.144 (4.150)	−0.0892 (0.298)	−8.575 (8.823)	Constant	−0.0359 (4.147)	−0.0794 (0.297)	−8.364 (8.814)
Individual fixed effects	Yes	Yes	Yes	Individual fixed effects	Yes	Yes	Yes
Time fixed effects	Yes	Yes	Yes	Time fixed effects	Yes	Yes	Yes
Observation	3,093	3,093	3,093	Observation	3,093	3,093	3,093
*R* ^2^	0.022	0.040	0.022	*R* ^2^	0.022	0.040	0.022

The symbols *, **, and *** denote significance at the 10, 5, and 1% significance levels, respectively.

Robust standard errors are presented in parentheses.

##### Model 3 regression with replacement of moderating variable

Yan and Jin (2014) believed that the higher the education level of executives in state-owned enterprises was, the stronger the fairness preference was. This article uses this index as a substitute variable for fairness preference of robustness tests, thus forming six groups of robustness test models of Model 3. The regression results are shown in Table 7:

**TABLE 7:** Robust test Model 3 with replacement of moderating variable.

Variables	Model 3-7 (*ROA*)	Model 3-8 (*EPS*)	Model 3-9 (*ROE*)	Variables	Model 3-10 (*ROA*)	Model 3-11 (*EPS*)	Model 3-12 (*ROE*)
*WDl*	0.0119*** (0.00387)	0.00111*** (0.000317)	0.0203** (0.00817)	*WDa*	0.0204*** (0.00704)	0.00207*** (0.000565)	0.0368** (0.0151)
*WDlsq*	−1.51e-05* (7.80e-06)	−1.36e-06* (8.10e-07)	−2.30e-05 (1.51e-05)	*WDasq*	−4.28e-05** (2.06e-05)	−4.88e-06** (2.06e-06)	−7.45e-05* (4.14e-05)
*BG*	0.0659 (0.525)	−0.0498 (0.0446)	−0.173 (1.084)	*BG*	0.0587 (0.531)	−0.0559 (0.0451)	−0.216 (1.100)
*BG* × *WDl*	−0.0157*** (0.00601)	−0.000772 (0.000529)	−0.0178 (0.0125)	*BG* × *WDa*	−0.0248** (0.0111)	−0.00144 (0.000965)	−0.0289 (0.0233)
*BG* × *WDlsq*	1.94e-05 (1.33e-05)	1.53e-06 (1.36e-06)	1.71e-05 (2.64e-05)	*BG* × *WDasq*	5.38e-05 (3.52e-05)	5.87e-06 (3.79e-06)	5.72e-05 (7.23e-05)
*LNN*	0.322 (0.436)	0.0504* (0.0266)	1.250 (1.020)	*LNN*	0.319 (0.436)	0.0492* (0.0267)	1.240 (1.016)
*POT*	0.0198 (0.0151)	0.00203* (0.00115)	0.0389 (0.0296)	*POT*	0.0198 (0.0151)	0.00203* (0.00114)	0.0389 (0.0293)
*POM*	0.0359** (0.0180)	0.00163 (0.00152)	0.0489 (0.0371)	*POM*	0.0352* (0.0179)	0.00158 (0.00150)	0.0478 (0.0369)
*PUT*	0.0188 (0.0179)	0.000753 (0.00112)	0.0288 (0.0327)	*PUT*	0.0189 (0.0179)	0.000749 (0.00111)	0.0286 (0.0327)
*PSS*	−0.0321* (0.0175)	−0.00177 (0.00132)	−0.0872** (0.0363)	*PSS*	−0.0316* (0.0175)	−0.00175 (0.00132)	−0.0867** (0.0363)
*TOP10*	−0.0335*** (0.00911)	−0.00348*** (0.000801)	−0.0599*** (0.0173)	*TOP10*	−0.0332*** (0.00914)	−0.00346*** (0.000800)	−0.0595*** (0.0173)
*BOD*	0.131 (0.178)	0.0118 (0.0162)	0.429 (0.368)	*BOD*	0.132 (0.177)	0.0119 (0.0161)	0.430 (0.367)
*IDP*	0.0341 (0.0383)	0.00188 (0.00293)	0.0878 (0.0764)	*IDP*	0.0321 (0.0384)	0.00172 (0.00292)	0.0846 (0.0764)
*DUAL*	0.319 (0.513)	0.0345 (0.0346)	0.808 (1.101)	*DUAL*	0.295 (0.512)	0.0296 (0.0344)	0.749 (1.094)
Constant	0.517 (4.162)	−0.0460 (0.301)	−7.526 (8.909)	Constant	0.608 (4.166)	−0.0257 (0.300)	−7.280 (8.875)
Individual fixed effects	Yes	Yes	Yes	Individual fixed effects	Yes	Yes	Yes
Time fixed effects	Yes	Yes	Yes	Time fixed effects	Yes	Yes	Yes
Observation	3,093	3,093	3,093	Observation	3,093	3,093	3,093
*R* ^2^	0.029	0.043	0.024	*R* ^2^	0.028	0.044	0.024

The symbols *, **, and *** denote significance at the 10, 5, and 1% significance levels, respectively.

Robust standard errors are presented in parentheses.

It is observed that the coefficient significance of the squared terms (*WDlsq* and *WDasq*), the one-degree terms (*WDl* and *WDa*) and their interaction terms (*BG* × *WDl* and *BG* × *WDa*) of the explanatory variable in Table 7 is not significantly different from that in Table 5. However, the coefficients of quadratic interaction terms (*BG* × *WDlsq* and *BG* × *WDasq*) are not significant in the six models but are still positive. At the same time, the coefficient directions of the above four terms are consistent with those in Table 5, indicating that the moderating effect of fairness preference *Z* is to weaken the relationship between the compensation gap within the TMT and corporate performance. However, the weakening effect is significant when the degree of external compensation inequity *F* is the fairness preference index, while the weakening effect of the education background *BG* index is less significant.

## Discussion

Previous studies on the influencing factors of corporate performance mainly focus on two parts: one is external factors, mainly including the degree of marketization (Dai and Guo, 2020), media attention (Bai et al., 2019), and government factors (Haider et al., 2018; Najaf and Najaf, 2021), legal factors (Trevlopoulos et al., 2021). The second is internal factors. It mainly includes organizational culture (Sari et al., 2018), capital structure (Uremadu and Onyekachi, 2018), executive characteristics (Leng and Kang, 2022), executive compensation (Rehman et al., 2021), and corporate characteristics (Richards et al., 2019; Younis and Sundarakani, 2019; Shahbaz et al., 2020). In contrast, external factors are difficult to control, while enterprises have more initiative in the improvement of internal factors. As a kind of special human capital in the enterprise, TMT has a great impact on corporate performance. As an incentive mechanism, the compensation gap within the TMT potentially affects the efforts of executives on corporate performance to a considerable extent.

The study focused on investigating the relationship between the compensation gap within the TMT and corporate performance through the moderating influence of fairness preference. Existing studies on the relationship between TMT and corporate performance have drawn inconsistent conclusions. Sun et al. (2020) found through empirical tests that the internal vertical compensation gap between CEOs and non-CEOs was positively correlated with corporate performance, the relationship between the internal horizontal pay gap within non-CEOs and corporate performance was inverse-U-shaped, and the degree of marketization strengthened the incentive effect of the vertical and horizontal pay gap. Li and Wang (2022) argued that when the CEO also served as the chairman of the board of directors, acting as the “single line liaison” between the board of directors and the enterprise, the compensation of the CEO was much higher than that of non-CEO executives and the CEO-TMT internal compensation gap was negatively correlated with the corporate performance. The increase in the compensation gap of the executive team can motivate executives to make innovative decisions and improve innovation performance (Hou, 2018). Mountouri (2019) explored the effect of the within-board compensation gap on the performance of the organization, the results suggested that the firm performance was affected positively by the compensation gap when measured as the Return on Assets, the Return on Equity, or Tobin’s Q.

The findings of the study model are consistent with the literature (Chen and Zhang, 2010; Huang, 2012; Chen et al., 2019; Fu et al., 2022). All these studies have proved the inverted U-shaped relationship between the compensation gap within the TMT and corporate performance. That is, there is a significant positive correlation between the optimal compensation gap. The larger the compensation gap, the better the corporate performance will be. This is consistent with the claims of tournament theory. But when the optimal compensation gap is exceeded, there is a significant negative correlation. The larger the compensation gap, the worse the corporate performance will be. This is in line with the inferences of equity theory. Different from previous studies, we further explore the role of fairness preference on the relationship between the compensation gap within the TMT and corporate performance based on social preference theory and conclude that fairness preference will weaken the correlation between the two.

The theoretical model analysis of this study believes that there is an optimal value of compensation gap in the traditional tournament model, and the tournament model based on the fairness preference of agents does not change this conclusion. The existence of the optimal value of the compensation gap indicates that the compensation gap is not the larger the better. In the empirical test part, through the regression method of two-way fixed effects, it is verified that there is a more significant inverted U-shaped relationship between the compensation gap within the TMT and corporate performance. The first hypothesis that there is an inverted U-shaped relationship between the TMT compensation gap and corporate performance is confirmed. That is, within the optimal value of the compensation gap, there is a significant positive correlation between them. The larger the compensation gap, the higher the corporate performance. When the optimal value is exceeded, there is a significant negative correlation between them. The larger the compensation gap, the lower the corporate performance.

In the theoretical model analysis part of the study, it is found that the existence and enhancement of fairness preference will reduce the marginal contribution of the compensation gap to corporate performance, that is, the incentive effect of the tournament will be reduced. Further analysis of the empirical regression results shows that the moderating effect of fairness preference on the relationship between the compensation gap within the TMT and corporate performance is as follows:

Within the optimal compensation gap, fairness preference will weaken the positive relationship between the compensation gap within the TMT and corporate performance. When it exceeds the optimal compensation gap, fairness preference will also weaken the negative relationship between the compensation gap within the TMT and the corporate performance. The second hypothesis that fairness preference moderates the correlation between the TMT compensation gap and corporate performance is supported, but the result goes in the opposite direction of Hypothesis 2b. When the optimal compensation gap is exceeded, fairness preference will not strengthen the negative relationship between them. On the contrary, fairness preference will weaken the relationship between them. This indicates that due to the attention of top management members to the fairness of compensation distribution results, the sensitivity of corporate performance to the compensation gap within the TMT is weakened.

## Conclusion

In this article, the fairness preference theory in behavior theory is introduced to the traditional tournament model, and a tournament model based on the fairness preference of agents is constructed, which is more in line with reality. Through the derivation and analysis of the theoretical model, and combined with the multivariate regression analysis of the panel data of 3,093 observations of 733 nonfinancial listed companies in Shanghai and Shenzhen stock markets from 2014 to 2020, this article discusses and tests the relationship between the compensation gap within the TMT and the corporate performance, and the moderating effect of fairness preference on the relationship between them.

The main conclusions are as follows: (1) There is an inverted U-shaped relationship between the TMT compensation gap and corporate performance. Within the optimal compensation gap, there is a significant positive correlation. The larger the compensation gap, the better the corporate performance will be. When the optimal compensation gap is exceeded, there is a significant negative correlation. The larger the compensation gap, the worse the corporate performance will be. (2) Fairness preference will weaken the correlation between the TMT compensation gap and corporate performance. Within the optimal compensation gap, the fairness preference will weaken the positive relationship between them, and when it exceeds the optimal compensation gap, the fairness preference will also weaken the negative relationship between them.

Combining the subject regression and robustness tests of this study, the measurement effects of the two empirical indicators of fairness preference are not the same. On the one hand, in terms of the moderating effect on the relationship between the compensation gap within the TMT and corporate performance, the degree of external compensation inequity is consistent with the effect direction of educational background. That ensures the robustness of the effect direction of fairness preference. On the other hand, compared with the degree of external compensation inequity, the moderating effect of educational background on the correlation between them is less significant. This shows that compared with the degree of external compensation inequity, the education background index is not an excellent substitute variable of fairness preference.

## Implications

### Managerial implications and policy suggestions

#### The design of compensation gap should include consideration of fairness preference of senior executives

The intensity of senior executives’ fairness preference affects the incentive effect of the compensation gap. The existence and enhancement of fairness preference will reduce the marginal contribution of the compensation gap to corporate performance. Fairness preference weakens the relationship between the compensation gap within the TMT and the corporate performance, this shows that due to the attention of senior executives to fairness, the tournament system can not fully play its original effectiveness. The effect of the compensation gap within the TMT on corporate performance is lower than that without fairness preference, and fairness preference will accelerate the emergence of the negative effect of the compensation gap. Therefore, when setting the compensation gap within the TMT, the enterprise should actively identify the strength of senior executives’ fairness preference, judge the strong degree of reaction of each top management member to the compensation inequality, and incorporate this into the consideration of setting the compensation difference and the compensation variation range within the same compensation level.

#### Be wary of the negative effect of the excessive compensation gap within the top management team

Based on the sample data, it is found that about 2% of the samples whose amount of the compensation gap within the TMT is too large, which has had a negative impact on their performance. Therefore, we recommend that these enterprises take measures to narrow the compensation gap between the ranks of their TMTs, in order to reduce the negative impact of the excessive compensation gap on corporate performance.

### Research limitations and prospects

First, fairness preference belongs to individual characteristics, which are heterogeneous and easily affected by the environment. The measurement of fairness preference is often seen in various experiments. The fairness preference in this study uses two indicators: the degree of external compensation inequity and educational background in the relevant literature. The former reflects the impact of the external environment, and the latter reflects individual heterogeneity. However, both indicators can only represent the intensity of preference, not the specific jealousy or sympathy of team members. In the future, indicators that can fully reflect fairness preference should be actively developed, or the combination mode of fairness preference indicators in experiments and large sample empirical regression should be actively explored.

Second, this study uses the unbalanced panel data of nonfinancial enterprises in the past 7 years for overall regression. Since the industry sample size of some non-manufacturing in the total sample is too small, the group regression by industry is not carried out. However, the compensation gap in each industry is different, and their respective optimal compensation gaps are likely to be different. For further study, we can increase the time span to expand the sample size to explore the differences between industries. In addition, the increase in time span is also helpful to explore the changes and impacts of fairness preference.

## Data availability statement

The original contributions presented in this study are included in the article, further inquiries can be directed to the corresponding author.

## Author contributions

XW and XY contributed to the conception and design of the study. XC and XY organized the database and performed the statistical analysis. XW and HZ performed original draft preparation, writing—review and editing. All authors contributed to the manuscript revision, read, and approved the submitted version.
